# Stress and Sleep Disorders in Polish Nursing Students During the SARS-CoV-2 Pandemic—Cross Sectional Study

**DOI:** 10.3389/fpsyg.2021.814176

**Published:** 2022-02-18

**Authors:** Iwona Bodys-Cupak, Kamila Czubek, Aneta Grochowska

**Affiliations:** ^1^Faculty of Health Sciences, Jagiellonian University Medical College, Kraków, Poland; ^2^Faculty of Health Sciences, University of Applied Sciences in Tarnow, Tarnow, Poland

**Keywords:** SARS-CoV-2, pandemic, sleep disorder, stress, student, coping

## Abstract

**Introduction:**

The world pandemic of the virus SARS-CoV-2 , which causes COVID-19 infection was announced by the World Health Organization (WHO) on March 11, 2020. Due to the restrictions that were introduced in order to minimize the spread of the virus, people more often suffer from stress, depression, anxiety, and sleep disorders. The aim of this study was evaluation of the stress levels and sleep disorders among nursing students during the pandemic SARS-CoV-2 .

**Materials and Study Methods:**

This is a cross-sectional study conducted among 397 nursing students on March 2020. The research tools used were original questionnaires, Perceived Stress Scale (PSS10), and the Athenian Insomnia Scale (AIS), as well as Coping with Stress Inventory (MiniCOPE).

**Results:**

Respondents felt a high level of stress, which occurred in 68.8% of interviewees regarding whether there was a danger of contracting COVID-19. Respondents experienced 84% stress levels when a family member suffered from COVID-19. Sleep disorders were determined mainly by the fear of infection and contact with someone who might be infected with the virus. Nursing students who felt a high level of stress often suffered from sleep disorders (70.2%) more frequently than students who felt a low or average stress level (30.4%). The respondents decided to cope with stress by denial, taking psychoactive substances, ceasing action, or blaming themselves. The greater the intensity of stress experienced by students, the more often they undertook avoidance behaviors or showed helplessness.

**Conclusion:**

During the pandemic, students experienced severe stress, which resulted in sleep disorders and avoidance behaviors.

## Introduction

The COVID-19 pandemic caused by the SARS-CoV-2 coronavirus has changed the lives of people around the world. The first cases caused by the new type of SARS-CoV-2 coronavirus appeared in the City of Wuhan in the Hubei province in China in November 2019. The virus turned out to be highly contagious and spread rapidly in many parts of the world reaching the size of a pandemic, announced by the World Health Organization (WHO) March 11, 2020 (World Health Organization, 2020). In Poland, the first case of coronavirus infection was confirmed on March 4, 2020.

On March 16, 2020, an epidemiological emergency was introduced in Poland, people were ordered to wear masks and maintain social distance. International air connections were suspended, shopping malls, schools, and universities were closed. The period of distance learning began. Nursing students who worked in hospitals at that time had to deal with a heavy burden. Most of the second-cycle students in Poland worked in health care units, primarily in hospitals. They had to combine work in difficult conditions and distance learning in the beginning of the second semester. The lack of effective medicine and vaccine has forced national governments to take radical measures. Most public institutions were closed, and restrictions in the form of lockdown and quarantine were introduced. This situation caused changes in the economic situation of the country (Czarkowski et al., 2020; Długosz, 2020; Kawska et al., 2020). The speed with which changes were introduced and the disinformation that occurred, negatively affected people's mental and physical health around the world. Sudden changes and uncertainty about what the future would bring undoubtedly contributed to people experiencing a significant degree of stress, as well as an increase in the incidence of sleep disorders, depression, and anxiety (Cellini et al., 2020; Czarkowski et al., 2020; Długosz, 2020; Mukhtar, 2020; Nwachukwu et al., 2020; Romero-Blanko et al., 2020; Shigemura et al., 2020; Vindegaard and Eriksen Benros, 2020; Xiao et al., 2020).

Stress in today's world is an integral part of life. This term can be used to describe not only sudden, traumatic experiences, but also the constant difficulties and changes that happen in life that are just part of living; the majority of society encounters and situations that cause stress on a daily basis and affect overall well-being (Bodys-Cupak et al., 2016, 2019a,b; Kowalczuk et al., 2017; Kwak et al., 2018; Mukhtar, 2020; Nwachukwu et al., 2020).

Seyle (1977) believed that no person in his life was able to free himself from stress. Stress is ubiquitous; therefore, there are many biological and psychological theories and definitions. It is very often analyzed in terms of a stimulus or reaction. Selye focused on the adverse effects of long-term stress and described the concept of “general adaptation syndrome,” in which he distinguished the phases of alarm, resistance, and exhaustion (Seyle, 1977). Selye divided stress into eustress and distress. He stated that stress does not have to be negative; it can be positive and even motivating. According to him, eustress is good, positive stress, which, in response to stressors, promotes the health of the body. Eustress is motivating and stimulating to development. On the other hand, distress is negative, difficult to control, and destructive to the organism (Seyle, 1977). According to Lazarus and Folkman (1984), stress in a psychological context is understood as the relationship between a person and the environment, which may be burdensome and threaten its well-being. The recognition of a particular relationship as stressful is determined by the individual cognitive assessment of the person participating in the relationship. The coping process is triggered in response to a stressful situation. It can be focused on an emotion or a problem (Lazarus and Folkman, 1984).

Due to its nature, a pandemic situation is a strong source of stress for people. The pandemic has changed the way we study and work. Medical personnel experience high levels of stress, anxiety, and sleep disorders (Kowalczuk et al., 2020; Rana et al., 2020; Xiao et al., 2020; Simonetti et al., 2021), as well as medical students, including nurses (Lund et al., 2010; Almojali et al., 2018; Aslan et al., 2020; Li et al., 2020; Aslan and Pekince, 2021; Dev Bhurtun et al., 2021). The increase in stress levels among nursing students due to COVID-19 may have various consequences. It can cause poor academic performance, lead to dropout, change in mental and physical health, and ultimately can affect the quality of care provided to patients (Wyatt et al., 2021).

The choice of coping methods depends on the assessment of the situation and may lead to a change of previous assessment. The concept of stress in the category of a stimulus is the concept of life changes, which is associated with a greater than usual mental and physical burden. In the concept of life changes, the environment in which the person experiences stress plays a significant role. Nursing students exhibit a variety of stress coping strategies, from active to avoidance (Bodys-Cupak et al., 2016, 2019a; McCarthy et al., 2018; Dev Bhurtun et al., 2021). Various factors also determine their coping strategies (Bodys-Cupak et al., 2021).

Stressogenic events negatively affect the body's immunity and may cause diseases (Kaczmarska and Curyło-Sikora, 2016). High-intensity chronic stress and not coping with it also negatively affects sleep quality.

Sleep is an essential factor when it comes to well-being as well as mental, physical, and cognitive functioning. After everyday activities, the body regenerates during sleep, ensuring later functioning (Troynikov et al., 2018). Slight shortening of sleep or its prolongation does not significantly affect physical and mental activity. However, if sleep is significantly shortened, it leads to disturbances in focus and deterioration of mood (Konturek, 2013; Troynikov et al., 2018). The importance of proper sleep has been confirmed in many areas of life, such as cognitive, emotional, social, and biological functioning. The role of sleep in the prevention of civilization diseases cannot be overlooked (Kaczor and Skalski, 2016). It is also noted that analyzing sleep problems becomes a useful indicator when it comes to assessing the risk of developing depression and the risk of relapse after remission (Heitzman, 2009).

More and more people are experiencing problems with sleep. Depending on the personality of the person and the accompanying diseases, 30–50% of people suffer from sleep disorders at some point in life (Pavlova and Latreille, 2018; Spielman et al., 2020).

Difficulty falling asleep causes anxiety, which makes the person agitated, which makes it difficult to fall asleep. It is a vicious cycle that leads to chronic insomnia, constant fatigue, and even symptoms of depression. There are many factors that influence insomnia and sleep disorders, such as age, stress, stimulants, exercise, and habits (Zhang et al., 2018; Spielman et al., 2020). Likewise, lack of exercise, low sun exposure, and increased use of electronic devices can also negatively impact sleep homeostasis (Cellini et al., 2020; Voitsidis et al., 2020). Previous studies suggest that pre-sleep negative cognitive activities following stressful events are associated with poor sleep activity (Takano et al., 2014).

Taking into account the fact that the effectiveness of actions taken depends on the intensity of the stress experienced by a person, it should be stated that the ability to deal with stress is an extremely important factor determining proper functioning. In the Polish literature, there are few results of studies on the impact of stress on coping and the occurrence of sleep disorders among nursing students during a pandemic. The results of the conducted research will allow the presentation of the issue in a different socio-cultural context.

The aim of the study was to assess the level of stress, sleep disorders, and ways of coping with the stress of Polish nursing students during the SARS-CoV-2 pandemic.

The hypothesis of this study is: The greater the severity of stress, the more frequent sleep disturbances occurred among nursing students during the SARS-CoV-2 pandemic.

## Materials and Methods

### Study Group Selection

At the design stage of the study, the criteria for inclusion in the study were established: participants needed to be studying nursing and able to give or obtain informed consent to participate in the study. The exclusion criterion was: individuals studying another medical field, and/or not having completed classes in a clinical ward. Participants were informed about the confidentiality and anonymity of the study and that participation was voluntary; also, that it was possible to withdraw/refuse to cooperate at any stage of the study. The participants were provided with information about the purpose of the study and given instructions on how to fill in the questionnaire. Completing the questionnaire was tantamount to giving consent to participate in the study.

The study was a cross-sectional study. It was carried out using the method of the diagnostic survey and method of estimation. The choice of doing a cross-sectional study was motivated by the fact that this type of study provides results in a relatively short time on a large group of respondents. The selection of the study group was purposeful. The study was developed and conducted in accordance with: (1) the principles of Good Scientific Practice; (2) the Act of May 10, 2018, on the protection of personal data; (3) the principles of the Helsinki Declaration; and (4) the Regulation of the European Parliament and the Council (EU) 2016/679 of April 27, 2016, on the protection of individuals with regard to the processing of personal data.

### Study Procedure

The study was conducted among nursing students on March 2020 using an online survey. The questionnaire was sent to 550 nursing students studying at the Institute for Health Protection of the State Higher Vocational School in Tarnów, and at the Faculty of Health Sciences at the Jagiellonian University Medical College in Krakow. Of the questionnaires distributed, 397 correctly completed questionnaires were received (72% of those distributed), 8 questionnaires were rejected due to significant data gaps that could distort the analysis. Submitting the completed questionnaires was tantamount to consent to participation in the study. In order to maintain anonymity, identification marks were provided on the research instruments. Sample size was calculated on G^*^Power 3.1.9.2, which revealed that a minimum of 305 participants were needed to perform an analysis with a significance level of 0.05 and a statistical power of 0.95.

## Questionnaires and Applied Measures

The study was carried out by applying the diagnostic survey method using the questionnaire technique. The research tools included the Perceived Stress Scale (PSS-10), the Athenian Insomnia Scale, the Coping with Stress Inventory (MiniCOPE), and the survey questionnaire developed by the authors of the study.

### Perceived Stress Scale

Perceived Stress Scale, PSS-10, was used to assess the intensity of stress related to the participant's own life situation over the last month. The authors of the tool are S. Cohen, T. Kamarck and R. Mermelstein. The tool was adapted to Polish conditions by Z. Juczyński and N. Ogińska-Bulik. The scale contained 10 questions on subjective feelings related to problems and personal events. For each question, the respondent could choose one of the indicated answers on a 5-point scale (0 – “never;” 1 – “almost never;” 2 – “sometimes;” 3 – “quite often;” 4 – “very often”). The score was obtained by adding up points where participants could get from 0 to 40 points. The score in the range from 1 to 4 sten is low, in the range from 7 to 10 sten—high, and in the range 5 and 6 sten—average. The higher the respondent's score, the greater the intensity of the stress. The internal reliability of the scale, determined using Cronbach's alpha, ranged from 0.72 to 0.90 (Juczyński and Ogińska-Bulik, 2012).

### The Athens Insomnia Scale

The Athens Insomnia Scale, AIS, is a self-descriptive tool for various symptoms of insomnia. It allows for assessing the quantitative measurement of insomnia symptoms taking into account the ICD-10 criteria. It consists of eight issues related to insomnia. Each criterion is assessed on a scale of 0–3 points. A score of 0–5 indicates a normal level, 6–10 indicates higher scores, and 11 or more suggests insomnia. The total score could reach 24 points. Validation in Polish was carried out by M. Fornal-Pawłowska, D. Wołyńczyk-Gmaj, and W. Szelenberger. The sensitivity of the scale is 93%, the specificity of the scale is 85%, and Cronbach's alpha coefficient was 0.88–0.91 (Fornal-Pawłowska et al., 2011).

### Brief COPE

Brief COPE was used to assess typical ways of reacting and feeling in severe stress and difficult situations. The tool by C. Carver, adapted by Z. Juczyński and N. Ogińska-Bulik, (called Inventory for Measuring Coping With Stress Mini-COPE) consisted of 28 statements, included in 14 strategies for coping with stress and difficult situations, which are divided into 7 factors, i.e., Active Coping, which included following strategies: Planning, Positive Reappraisal; Seeking Support, which on the other hand includes: Seeking Emotional Support and Seeking Instrumental Support; Helplessness, which consists of the Use of Psychoactive Substances, Suppression of Activities, Self-Blame; and Avoidance Behaviors, which include: Dealing with Something Else, Denial, Venting of Emotions. On the other hand, the strategies of Turning to Religion, Acceptance, and Sense of Humor were independent factors. For each statement, the respondents marked one of four possible answers: from “I almost never do this” (0 points) to “I almost always do this” (3 points). The score was calculated separately for each strategy, and the higher the score, the more often the strategy was used. In the in-depth analysis of the scores, problem-focused strategies were distinguished, including Active Coping, Planning, Seeking Instrumental Support, and strategies focused on emotions, including Seeking Emotional Support, Turning to Religion, and Denial. For example statements like *My efforts are focused on doing something about the situation* and *I am taking action to improve this situation* were included to Active Coping. Statements like *I am trying to work out a strategy or plan outlining what to do* and *I am seriously wondering what steps should be taken* were included to Planning. The reliability of the original version of the Inventory determined using Cronbach's alpha ranged from 0.62 to 0.89 (Juczyński and Ogińska-Bulik, 2012). Before using the questionnaires, the researchers obtained permission to use them.

The original questionnaire of the survey contained 12 questions: socio-demographic questions regarding, among others, gender, age, and year of study, as well as the COVID pandemic (e.g., work in a hospital dedicated to patients suffering from COVID-19; contact family member who takes care of a patient suspected of being infected with COVID-19, obligation to be on quarantine, obligation to use personal protective equipment). It made it possible to collect information that was used for an in-depth characterization of the study group and the assessment of factors influencing the perceived level of stress and insomnia.

## Statistical Methods

During the analysis of the collected research materials, statistical methods were also used, allowing for the development of results and conclusions. Correlations between quantitative variables were analyzed using the Spearman's rank correlation coefficient or the Pearson's χ^2^-test of independence. The strength of dependence was interpreted according to the following scheme: |*r*| ≥ 0.9—very strong dependence; 0.7 ≤ |*r*| <0.9—strong dependence; 0.5 ≤ |*r*| <0.7—moderately strong dependence; 0.3 ≤ |*r*| < 0.5—weak correlation; |*r*| < 0.3—very weak relationship (negligible).

The collected data was entered into Microsoft Excel and subjected to statistical analysis, for which the Pearson test of independence was used. The level of significance was set at *p* < 0.05. The calculations were made using SPSS 20 software.

## Results

### Study Group

Most of the study group of 397 students were between 20 and 30 years of age (*N* = 69.3%). Of respondents, 9.1% (*N* = 36) were 31 and 40 years of age, and 15.6% of respondents (*N* = 62) were between 41 and 50 years of age. The age group of 51–60 years was 6.0% (*N* = 24). The study group consisted of more women than men, with 93.7% female respondents (*N* = 372) and 6.3% male respondents (*N* = 25).

First-year student made up 49.9% of the respondents (*N* = 198), and 50.1% (*N* = 199) were second-year students.

Most of the surveyed students (*N* = 301, i.e., 75.8%) were full-time students. Every fourth respondent (*N* = 96, i.e., 24.2%) studied at part-time studies.

Recently, 11.6% of respondents (*N* = 46) have been quarantined due to the spread of COVID-19.

Every fourth respondent (*N* = 102, i.e., 25.7%) admitted that someone from his or her immediate family was quarantined. A group of 59.4% of students (*N* = 236) admitted that they had a health care professional in their immediate family. Work in a hospital dedicated to patients suffering from COVID-19 was performed by 12.6% of students (*N* = 50). According to 9.3% of students (*N* = 37), someone in their family worked in a hospital dedicated to COVID-19 patients. According to 15.9% of respondents (*N* = 63), someone from their family was taking care of a patient suspected of being infected with COVID-19.

The COVID-19 cases among family members were indicated by 6.3% of respondents (*N* = 25). The majority of people (*N* = 321, i.e., 80.9%) indicated that there were no cases of the virus in their families. Of the respondents, 0.8% (*N* = 3) suffered from COVID-19; 39.8% of respondents (*N* = 158) dealt with patients suspected of having COVID-19 in their work; 46.6% of people (*N* = 185) did not come into contact with such patients; and 13.6% (*N* = 54) did not know if they had contact with patients suspected of having COVID-19. Facemasks were the most frequently mentioned PPE (personal protective equipment) used in the workplace (*N* = 344, 86.6%). Almost half of the respondents (*N* = 193, 48.6%) used face shields, and more often than every fourth person (*N* = 104, 26.2%) used overalls. Gloves were rarely used (*N* = 47, 11.8%), and 12.1% of the respondents did not use any protection (*N* = 48); Of the participants, 79.6% (*N* = 316) indicated the implementation of procedures in the workplace for the treatment of a patient suspected of COVID-19; 68.8% of respondents (*N* = 273) feared contracting COVID-19; 20.4% of respondents (*N* = 81) were not afraid of developing this disease; and 10.8% of respondents (*N* = 43) did not think about it.

Most of the respondents (*N* = 261, 65.7%) took showers, washed their clothes, and disinfected their hands and products after returning from work; 19.9% of the respondents (*N* = 79) avoided contact with their relatives after returning from work; 8.3% (*N* = 33) of people lived away from home to avoid the risk of their family contracting COVID-19; and 6.0% of respondents (*N* = 24) did not answer the question about the protection of the family against transmission of the virus.

Based on the PSS-10 scale, it was found that 8.6% of the respondents (*N* = 34) experienced a low level of stress. The average level of stress was found in 25.4% of the respondents (*N* = 101). Most of the respondents (*N* = 262, 66.0%) experienced a high level of stress.

The results of the Athenian Insomnia Scale showed that 43.3% of respondents had no sleep disorders (*N* = 172). Sleep disturbances considered as normal occurred in every third person (*N* = 132, 33.2%). The probability of insomnia was 23.4% of the respondents (*N* = 93).

### Factors Influencing the Level of Perceived Stress

A high level of stress was demonstrated among 64.4% of respondents aged 20–30 and 69.7% of people over 30 (χ^2^ = 1.061; *p* = 0.3029). Higher levels of stress were more common in women (66.7%) than in men (56.0%) (χ^2^ = 1.188; *p* = 0.2758).

The level of perceived stress did not significantly depend (χ^2^ = 0.842; *p* = 0.3589) on the year of study. Slightly more students in the 1st year (68.2%), than the 2nd year (63.8%), assessed their stress level as high. Part-time students experienced a high level of stress more often than part-time students (74.0%) than full-time students (63.5%) (χ^2^ = 3.578; *p* = 0.0586). It was shown that high levels of stress were more common among students whose closest family members were quarantined due to contact with someone suffering from COVID-19 (74.5%; *p* = 0.0352). The level of stress experienced by the respondents was not related to the medical profession performed by a family member of the respondent (χ^2^ = 1.284; *p* = 0.2571).

High levels of stress were experienced more often by people who worked in a hospital dedicated to patients suffering from COVID-19 (78.0%) than other respondents (64.3%) (χ^2^ = 3.674; *p* = 0.0553). This relationship (although not statistically significant) also occurred when someone from the family of the respondents worked in a hospital dedicated to patients suffering from COVID-19 (78.4%) than in people who did not have such relatives in their family (64.7%) (χ^2^ = 2.788; *p* = 0.0950).

A high level of perceived stress was significantly more frequent in students who had in their immediate family people taking care of patients suspected of having COVID-19 (82.5%) than in other respondents (17.5%) (χ^2^ = 9.134; *p* = 0.0025).

In the families of 25 students surveyed, a family member suffered from COVID-19. Respondents (84.0%) whose families had contracted COVID-19 experienced high levels of stress more often than respondents (χ^2^ = 4.243; *p* = 0.1199) whose family members had not contracted the virus. It has not been shown, however, that the fact that the surveyed students had COVID-19 had an impact on the level of stress they felt.

It was shown that having COVID-19 did not significantly affect the level of perceived stress by the respondents (χ^2^ = 0.001; *p* = 0.9803).

Respondents who came into contact with patients suspected of having COVID-19 (75.3%), more often had a high level of stress than respondents (58.4%) (χ^2^ = 10.933; *p* = 0.0042) who had not come into contact with patients suspected of having COVID-19.

High intensity of perceived stress was more common among people who had procedures for dealing with a patient suspected of having COVID-19 (68.0%) in the workplace compared to other respondents (58.0%) (χ^2^ = 2.881; *p* = 0.0897).

People who were afraid of contracting COVID-19 (71.8%) more often than other respondents (51.9%) declared a high level of perceived stress (χ^2^ = 13.298; *p* = 0.0013).

Students who scored high on the perceived stress scale were more likely to protect their families from the transmission of the virus in various ways. Respondents with low/average stress levels more often did not answer the question about the protection of loved ones, which suggests that they did not implement any measures in this direction (χ^2^ = 17.374; *p* = 0.0006).

### Factors Influencing the Level of Insomnia

The occurrence of sleep disorders was significantly more frequent in people over 30 years of age (67.2%) compared to respondents ages 20–30 (52.0%) (χ^2^ = 7.965; *p* = 0.0048). The sex of the respondents did not significantly affect the occurrence of sleep disorders (χ^2^ = 0.237; *p* = 0.6260). It was not found that the occurrence of sleep disorders in the respondents differed significantly depending on the year of study (χ^2^ = 0.202; *p* = 0.6534). Both among the surveyed students of the 1st and 2nd year, the probability of sleep disorders occurred in a similar percentage of cases (55.6 and 57.8%, respectively). The occurrence of sleep disorders was more frequent among part-time students (72.9%) than full-time students (51.5%) (χ^2^ = 13.603; *p* = 0.0002).

It was not found that the occurrence of sleep disorders in the respondents was significantly related to someone from the immediate family of the respondent being quarantined due to contact with a person suspected of having COVID-19 (χ^2^ = 0.944; *p* = 0.3313).

Sleep disturbances were more common in people (60.6%) who had healthcare professionals in the family than the other respondents (50.9%) (χ^2^ = 3.638; *p* = 0.0565). It was noticed that sleep disturbances occurred more often in students who worked in a hospital dedicated to patients suffering from COVID-19 (68.0%) than in other respondents (55.0%) (χ^2^ = 2.988; *p* = 0.0839).

The respondents who had a family member working in a hospital dedicated to patients suffering from COVID-19 more often (73.0%) than the rest (55.0%) declared sleep disorders (χ^2^ = 4.414; *p* = 0.0356). Taking care of a patient suspected of having COVID-19 by a family member did not significantly affect students' occurrence of sleep disorders (χ^2^ = 0.405; *p* = 0.5247).

The occurrence of COVID-19 cases among family members did not significantly affect the sleep disorders of the respondents (χ^2^ = 1.540; *p* = 0.4630).

Having COVID-19 by the respondent did not significantly affect the occurrence of sleep disorders (χ^2^ = 0.123; *p* = 0.7259).

Sleep disturbances were more common among people who came into contact with patients suspected of having COVID-19 (66.5%) and people who did not know whether they had contact with such patients (63.0%) (χ^2^ = 14.846; *p* = 0.0006).

The implementation of procedures in the workplace for taking care of a patient suspected of having COVID-19 did not significantly affect the occurrence of sleep disorders in the respondents (χ^2^ = 2.204; *p* = 0.1377).

It was found that people who were afraid of contracting COVID-19 had sleep disorders more often (65.9%) than the rest of the respondents (37.0%) (χ^2^ = 30,569; *p* < 0.0001).

The occurrence of sleep disorders in the respondents was not significantly related to their protection of the family against virus transmission (χ^2^ = 4.345; *p* = 0.2266).

### The Influence of the Perceived Intensity of Stress on the Level of Insomnia

**Figure 1:**
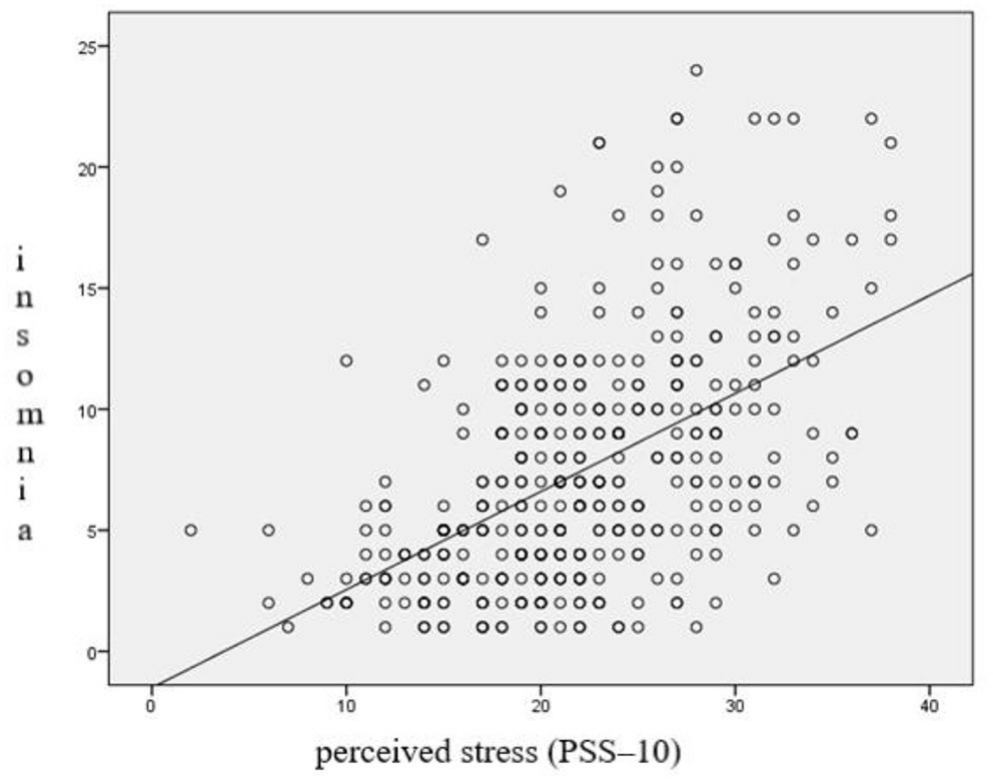
Perceived stress level (PSS-10) and sleep disorders—insomnia (AIS).

Students who felt stressed at a high level had more sleep disorders (70.2%) than those who experienced stress at a low or average level (30.4%). The differences were statistically significant (χ^2^ = 57.645; *p* < 0.0001) (Table 1; Figure 1).

**Table 1:** Occurrence of sleep disorders and the level of perceived intensity of stress.

			**Level of stress (PSS-10)**		**Total**
			**Low/average**	**High**	
The Athens Insomnia scale	Norm	*N*	94	78	172
		%	69.6%	29.8%	43.3%
	Borderline norm/probable insomnia	*N*	41	184	225
		%	30.4%	70.2%	56.7%
Total		*N*	135	262	397
		%	100.0%	100.0%	100.0%

*χ^2^ = 57,645; p < 0.0001.*

*χ^2^, Pearson test; p, significance level*.

The higher the intensity of the stress experienced by the respondents, the more often the respondents decided to cope with denial, taking psychoactive substances, ceasing action, or blaming themselves. The higher that the stress level was, the more often helplessness and avoidance behavior were chosen rather than active coping, strategies for seeking support, or sense of humor. Similarly, the more often the respondents experienced sleep disorders, the less often they undertook active forms of coping with stress and more often they chose avoidance behaviors (Table 2).

**Table 2:** The level of perceived stress and sleep disorder and coping with stress of nursing students.

	**The level of perceived stress (PSS-10) (0–40 points)**		**The Athens Insomnia scale (AIS) (0–24 points)**	
	**Rho**	***p***	**Rho**	***p***
**Mini-COPE detailed scales**				
Active coping	−0.093	0.0653	−0.045	0.3689
Planning	−0.029	0.5692	−0.056	0.2689
Positive reappraisal	−0.174	**0.0005**	−0.177	**0.0004**
Acceptance	0.006	0.9093	−0.068	0.1792
Sense of humor	−0.127	**0.0112**	−0.186	**0.0002**
Turning to religion	0.071	0.1567	0.056	0.2652
Seeking emotional support	−0.088	0.0807	−0.055	0.2741
Seeking instrumental support	−0.019	0.7025	0.016	0.7494
Dealing with something else	0.060	0.2354	−0.007	0.8888
Denial	0.219	**0.0000**	0.194	**0.0001**
Venting of emotions	0.246	**0.0000**	0.126	**0.0117**
Use of psychoactive substances	0.141	**0.0050**	0.173	**0.0005**
Activities cessation	0.202	**0.0000**	0.147	**0.0034**
Self-blame	0.285	**0.0000**	0.232	**0.0000**

*Rho, Spearman correlation; p, significance level. Bold values p value–level of statistical significance*.

## Discussion

The emergence of the new SARS coronavirus-CoV2 has increased the level of stress perceived by people and revealed the need to treat the psychological aspects of epidemics and pandemics and to treat these events as phenomena that have psychological effects (Aslan and Pekince, 2021). Students and health care workers were the most exposed to stress (Simionescu et al., 2021).

They were concerned about their health as well as the health of their family members. In turn, the intensification of stress contributed to the occurrence of sleep disorders. It is noticeable in the results of this study.

A high level of stress was demonstrated among respondents. Similar results were achieved by Aslan and Pekince (2021). In a study conducted among Turkish nursing students, the results show that they experienced high levels of stress, mild anxiety, and moderate depression, with 5.6% reporting low levels of perceived stress, 23% moderate, and 71.2% high (Aslan and Pekince, 2021). A similar relationship with regard to gender has also been shown. More often, female students reported significantly higher levels of stress compared to male students (Andrzejewska et al., 2018; Becker et al., 2018; Piotrowski et al., 2020; Aslan and Pekince, 2021; Simionescu et al., 2021).

The results of other studies by Xiong et al. (2020), and other co-authors as well as Lai et al. (2020) also prove that women are more prone to higher levels of stress than men. It was noticed that the stress level varied and depended on the student's status, the number of days when the university was closed, the occurrence of unemployment, the occurrence of infections in close surroundings, and low self-evaluation of health (Xiong et al., 2020).

While analyzing the literature and comparing the results, an article was found on the impact of lockdown on the quality of sleep in students and administrative staff. Research by Marelli et al. (2021) , and other authors indicated that the lockdown period in the SARS-CoV-2 pandemic contributed to the deterioration of sleep quality. Problems with falling asleep, maintaining sleep, and its efficiency were mainly influenced by the risk of developing COVID-19. Difficulties with falling asleep occurred in 55% of students, and 30% of respondents had problems waking up early. It was also noticed that 34.3% of the nursing students developed symptoms of depression. In the study, women were found to be more prone to sleep disorders, but taking into account only students, no significant differences related to sleep disorders were found (Marelli et al., 2021). A study by Romero-Blanko et al. (2020) similarly showed that the lockdown period resulted in poorer sleep quality (with the exception of sleep duration). Moreover, it turned out that the decreased quality of sleep was found in people who reported problems with anxiety or depression and those who did not. The study by Xiao et al. (2020) showed that the sleep quality of the respondents in central China during the pandemic decreased in people with higher levels of perceived stress. The results of studies by Simonetti et al. (2021) carried out during the pandemic among Italian nurses showed a significant correlation between perceived anxiety and decreased quality of sleep.

The impact of stress on students' sleep is also confirmed by the studies of other authors (Lund et al., 2010). The study by Almojali et al. (2018), documents a high prevalence of stress and poor sleep quality in a sample of Saudi medical school students (Almojali et al., 2018). Similarly, the results of Lai et al. (2020) showed that full-time international university students experienced more intense stress and insomnia during a pandemic. The analysis of sleep disorders among respondents of the study by Piotrowski et al. (2020) showed that they occur in both women and men, but women are more prone to sleep problems. Similar results have been shown by this research conducted among nursing students.

In China, where people were the first to experience SARS-CoV-2 infections in the world, a mental health study was conducted among Chinese students. The incidence of stress, depression, and anxiety was 35.1, 22.4, and 12.1%, respectively (Li et al., 2020).

This study similarly observed that the higher levels of stress concerned women, and it was higher if the SARS-CoV-2 concerned someone from their nearest family. Sleep disorders appeared when someone in the family worked with SARS-CoV-2 patients, and the students worked in a hospital dedicated to COVID-19 patients. The vast majority did not complain about sleep disorders if someone in the family was in quarantine or when a family member was taking care of someone with COVID-19. It will be a purposeful task to look at people who suffer from stress at a very high level. The relationship between sleep disorders and the intensity of perceived stress was also confirmed in studies where it was additionally established that the effect of stress on sleep difficulties is strengthened by meditation and/or negative affects (Amaral et al., 2018).

The higher the stress level was, the more often helplessness and avoidance behaviors were chosen rather than active coping, strategies for seeking support, or sense of humor in own study. In the study of Simionescu et al. (2021), the most effective coping strategies against stress for nurses during the pandemic were based on self-control and spiritual dimension (Simionescu et al., 2021). In a study by Lai et al. (2020), the top three most commonly used coping strategies among students during the COVID-19 pandemic were listening to music (78%), eating, or cooking (66%), and video or mobile gaming.

One an article regarding students' coping strategies during the COVID-19 pandemic found that ~35% of students experienced some level of anxiety and used four types of coping strategies: seeking social support, avoidance/acceptance, mental disengagement (Khoshaim et al., 2020). Before the pandemic, authors found that the most common coping behavior used by nursing students was transference, followed by remaining optimistic, and problem-solving, while the least used was avoidance (Zhao et al., 2015; Bodys-Cupak et al., 2016). Specific strategies of coping with stress are necessary for those nursing students who are preparing for future jobs, to safely care for their patients.

During the SARS pandemic in 2003, fear of infection was reported to have had negative psychological effects (Bai et al., 2004; Chua et al., 2004; Lee et al., 2007), so it seems appropriate to conduct research to determine the best ways to ensure safety and counteract PTSD (post-traumatic stress disorder). We should remember that students are important members of our health system; they are essential for the health of the patient, and possible future pandemics. It is important to support future medical personnel in creating the most effective stress-coping strategies. Today, the neglect of the needs related to maintaining mental comfort may result in increasing disorders that appear over the next years.

### Limitation of the Study

The main limitations of the study are related to areas of data collection methods. Data collection was undertaken at one point in time rather than longitudinally. The characteristics of the participants—young people, mainly women—could represent a bias.

The selection of the group of respondents was deliberate. The research was carried out using nursing students from only two universities, thus the conclusions cannot be applied to the general population. More research is needed.

### Implications to Practice

Nursing students in the face of a pandemic, like medical personnel, experience stress and are at risk of developing disorders, especially in the sphere of mental functioning. Therefore, it is important to quickly assess and recognize the individual's situation and needs in order to provide adequate support and shape active forms of coping with stress. Greater psychological support from academic institutions is needed to enhance female students' mental health and resilience. This will save students from serious consequences, such as developing depression or post-traumatic stress disorder. These findings are important for both nursing schools and hospitals.

## Conclusion

1. During the pandemic, students experienced severe stress, which resulted in sleep disorders. The greater the intensity of stress experienced by students, the more often they undertook avoidance behaviors or showed helplessness.
2. The intensity of perceived stress was higher in students if there was a risk of contact with a sick or quarantined person, or the respondent worked in a hospital dedicated to patients suffering from COVID-19.
3. Sleep disorders intensified if the respondents had health care workers in their families, and they occurred in students who worked in a hospital dedicated to patients suffering from COVID-19.

## Data Availability Statement

The raw data supporting the conclusions of this article will be made available by the authors, without undue reservation.

## Ethics Statement

The research was approved by the Bioethics Committee in Tarnów– No. of approval: 2/9277/2019. Additionally, the patients/participants provided their written informed consent to participate in this study. The study was developed and conducted in accordance with the protocols accept by the university authorities' and the principles of Good Scientific Practice; the Act on the protection of personal data; the principles of the Helsinki Declaration; and the Regulation on the protection of individuals with regard to the processing of personal data.

## Author Contributions

IB-C and AG: conceptualization, data curation, formal analysis, funding acquisition, and methodology. IB-C, AG, and KC: investigation and writing— review and editing. IB-C: supervision and writing—original draft. All authors have read and agreed to the published version of the manuscript.

## Conflict of Interest

The authors declare that the research was conducted in the absence of any commercial or financial relationships that could be construed as a potential conflict of interest.

## Publisher's Note

All claims expressed in this article are solely those of the authors and do not necessarily represent those of their affiliated organizations, or those of the publisher, the editors and the reviewers. Any product that may be evaluated in this article, or claim that may be made by its manufacturer, is not guaranteed or endorsed by the publisher.
